# Biological Degradation of Aflatoxin B_1_ by Cell-Free Extracts of *Bacillus velezensis* DY3108 with Broad PH Stability and Excellent Thermostability

**DOI:** 10.3390/toxins10080330

**Published:** 2018-08-14

**Authors:** Xian Shu, Yuting Wang, Qing Zhou, Minghao Li, Hao Hu, Yuhan Ma, Xue Chen, Jun Ni, Weiwei Zhao, Shengwei Huang, Lifang Wu

**Affiliations:** 1 Key Laboratory of High Magnetic Field and Ion Beam Physical Biology, Hefei Institutes of Physical Science, Chinese Academy of Sciences, Hefei 230031, China; sx360775419@gmail.com (X.S.); hsw-itb@hotmail.com (Y.W.); USTCzq1@outlook.com (Q.Z.); limh@ipp.ac.cn (M.L.); huhaoasd@mail.ustc.edu.cn (H.H.); yuhanmie@ipp.ac.cn (Y.M.); chenxueahau@163.com (X.C.); nijun@ipp.ac.cn (J.N.); annyzhao@ipp.ac.cn (W.Z.); 2 School of Life Sciences, University of Science and Technology of China, Hefei 230026, China; 3 The Sericultural Research Institute, Anhui Academy of Agricultural Science, Hefei 230031, China; 4 Key Laboratory of Environmental Toxicology and Pollution Control Technology of Anhui Province, Hefei Institutes of Physical Science, Chinese Academy of Sciences, Hefei 230031, China

**Keywords:** mycotoxin, aflatoxin B_1_, *Bacillus velezensis*, biodegradation, detoxification

## Abstract

(1) Background: Aflatoxin contamination in food and grain poses serious problems both for economic development and public health protection, thus leading to a focus on an effective approach to control it; (2) Methods: Aflatoxin B_1_ (AFB_1_) degrading bacteria were isolated using a medium containing coumarin as the sole carbon source, and the biodegradation of AFB_1_ by the isolate was examined by high performance liquid chromatography, and liquid chromatography mass spectrometry; (3) Results: a bacterial strain exhibiting strong AFB_1_ degradation activity (91.5%) was isolated and identified as *Bacillus velezensis* DY3108. The AFB_1_ degrading activity was predominantly attributed to the cell-free supernatant of strain DY3108. Besides, it was heat-stable and resistant to proteinase K treatment but sensitive to sodium dodecyl sulfate treatment. The optimal temperature for the maximal degradation of AFB_1_ was 80 °C. Even more notable, the supernatant showed a high level of activity over a broad pH (4.0 to 11.0) and exhibited the highest degradation (94.70%) at pH 8.0. Cytotoxicity assays indicated that the degradation products displayed significantly (*p* < 0.05) lower cytotoxic effects than the parent AFB_1_; (4) Conclusions: *B. velezensis* DY3108 might be a promising candidate for exploitation in AFB_1_ detoxification and bioremediation in food and feed matrices.

## 1. Introduction

Aflatoxins are a group of toxic secondary metabolites primarily synthesized by filamentous fungal species including *Aspergillus flavus* and *Aspergillus parasiticus* in both field and storage conditions [1,2]. Aflatoxin B_1_, B_2_, G_1_ and G_2_ (AFB_1_, AFB_2_, AFG_1_ and AFG_2_) are four major aflatoxins, and AFB_1_ has been classified as a Group I naturally occurring carcinogen due to its hepatotoxic, carcinogenic, teratogenic, and immunosuppressive characteristics [3,4]. Furthermore, high aflatoxin B_1_ contamination in food can occur in the tropical region where fungal growth and proliferation are favored by high temperatures and humidity, and thus has attracted worldwide attention [5,6].

The prolonged contamination of agricultural and food products by aflatoxins has proposed an emergent demand to detoxify contaminated food and feed using different methods. Although several physical and chemical strategies have been proposed to degrade AFB_1_ [7,8,9], limitations such as not providing the desired efficacy, safety and nutrient retention along with cost requirements have made them less desirable [10,11]. However, as valuable alternatives to physicochemical methods, biological degradation of AFs are attracting substantial attention due to their additional benefits such as their minimal loss of product qualities, safety, efficiency, economic and eco-friendly nature [12,13].

There are two key directions in control aflatoxin contamination: preventing the growth of toxigenic *A. flavus*, namely prevention and if contamination occurs, then detoxify aflatoxin-contaminated commodities by removing the toxic compounds [14]. Over the past decades, several bacterial and fungal species have been exploited to manage aflatoxin contamination, including non-aflatoxigenic strains of *A. flavus* [15], saprophytic yeasts [16], *Mycobacterium fluoranthenivorans* [17], *Rhodococcus erythropolis* [18], *Pseudomonas putida* [2], *Pontibacter* sp. [5], *Streptomyces* sp. [19], and *Rhodococcus pyridinivorans* [20]. In addition, *Bacillus* spp. also appeared as valuable candidates for controlling filamentous fungal growth and inhibiting mycotoxin production [14]. *Bacillus subtilis* UTBSP1 can effectively restrict the growth of *A. flavus* growth and remove AFB_1_ in pistachio nut as previously described by Farzaneh et al. [21], while 67.2% AFB_1_ degradation by cell-free supernatant of *Bacillus subtilis* JSW-1 was also observed by Xia et al. [14]. Raksha et al. [22] reported that *Bacillus licheniformis* CFR1 can reduce AFB_1_ by 94.7% and eliminate the AFB_1_ induced mutagenicity, indicating that *Bacillus* spp. might be excellent candidates in the field of food safety. Despite these fungi and bacteria were able to degrade AFB _1_ effectively, few strains have been applied commercially because the actual use of these microorganisms or their metabolites were affected by the long reaction times, narrow working temperatures, or relatively low degradative efficiency. Therefore, exploring microorganisms or their metabolites that degrade and detoxify AFB_1_ with excellent degradation efficacy, wide temperature ranges, or short degradation times would be highly beneficial [23].

In this study, we isolated a new *Bacillus* bacterium from the soil that exhibited high AFB_1_ degradation activity with broad pH tolerance and excellent thermostability. The optimal degradation conditions of AFB_1_ by the strain were determined, and the cytotoxic potential of the metabolites formed after degradation was also analyzed.

## 2. Results and Discussion

### 2.1. Isolation and Identification of AFB_1_-Degrading Bacteria

**Figure 1:**
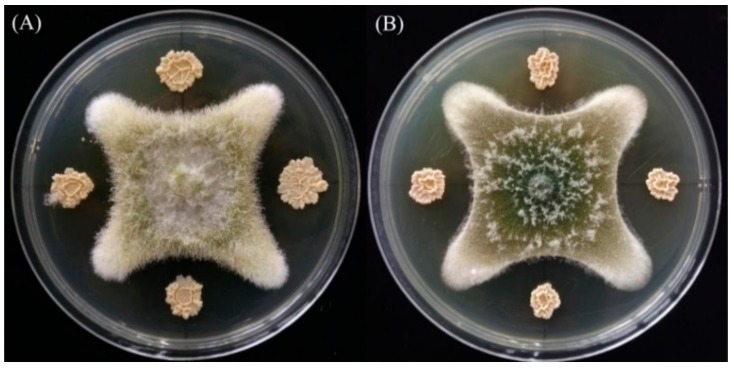
In vitro inhibition of isolate DY3108 on *Aspergillus flavus* (**A**) and *Aspergillus parasiticus* (**B**) growth. Agar plug (5 mm diameter) from *A. flavus* or *A. parasiticus* was placed in the center of a potato dextrose agar (PDA) plate, and isolate DY3108 was cultured on the plate at four equidistance sites 3 cm apart from the center.

As the basic molecular structure of all aflatoxins, coumarin is considered to be a feasible, affordable, and effective tool to select AFB_1_-degrading microorganisms [24]. In this study, coumarin was used as the sole carbon source in the preliminary screen of bacteria with AFB_1_-degrading ability, and 13 bacteria were isolated from the soil samples. However, secondary screening results showed that strain DY3108 possessed the highest degradation rate of 91.5% after 96 h incubation at 30 °C. In addition, an in vivo antagonistic effect test showed that strain DY3108 could significantly reduce the mycelial growth of *A. flavus* and *A. parasiticus* (Figure 1). The greatest amount of degradation of AFB_1_ (66.43%) was recorded in the maize containing co-cultures of strain DY3108 and *A. flavus* strain 3.6305 (Table 1). Thus, this isolate was chosen for further study.

**Figure 2:**
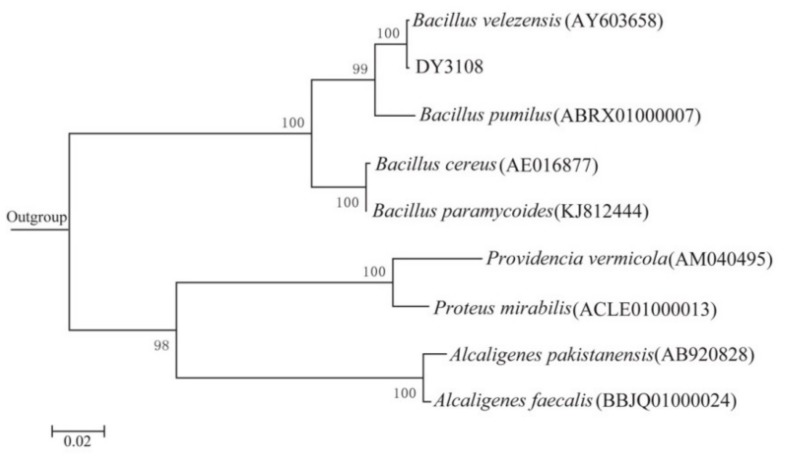
Phylogenetic tree built using Maximum likelihood method based on the 16S rRNA gene sequence of strain DY3108 and the sequences of representative strains from GenBank. The bar represents 0.02 substitutions per site. *Sphingobacterium zeae* (KU201960) was used as an outgroup.

According to 16S rRNA gene sequence and phylogenetic evolution analysis, the closest relative of isolate DY3108 was *Bacillus velezensis* CR-502(T) with 99% similarity (Figure 2). In addition, strain DY3108 showed the typical characteristics of *Bacillus* sp. (Table S1). Therefore, the strain DY3108 identified as *B. velezensis* DY3108. *B. velezensis* CR-502(T) is a gram-positive, endospore-forming bacterium that can produce surfactant molecules [25]. In addition, several *Bacillus* species, such as *Bacillus subtilis* JSW-1 [14] and *B. licheniformis* CFR1 [22], have been reported to possess aflatoxins degrading capacity. However, this is the first study that showed that a *B. velezensis* strain could remove more than 91% of AFB_1_ from the liquid media.

### 2.2. AFB_1_ Degradation by B. Velezensis DY3108

**Figure 3:**
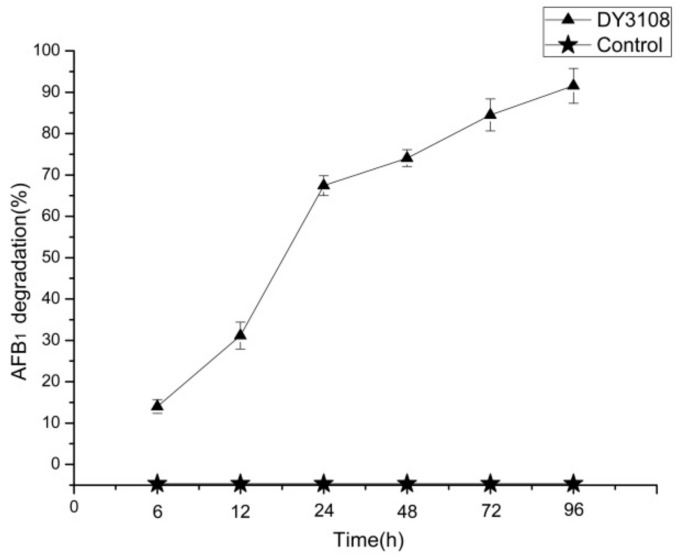
Kinetics of the Aflatoxin B_1_ (AFB_1_) degradation of by *B. velezensis* DY3108 over 96 h at 30 °C.

As observed by high-performance liquid chromatography (HPLC), *B. velezensis* DY3108 degraded AFB_1_ in a continuous process, and more than 90% of AFB_1_ was degraded in 96 h, while AFB1 concentration in the control broth was remaining stable from 0 to 96 h, with no significant difference (*p* < 0.05) observed (Figure 3).

**Figure 4:**
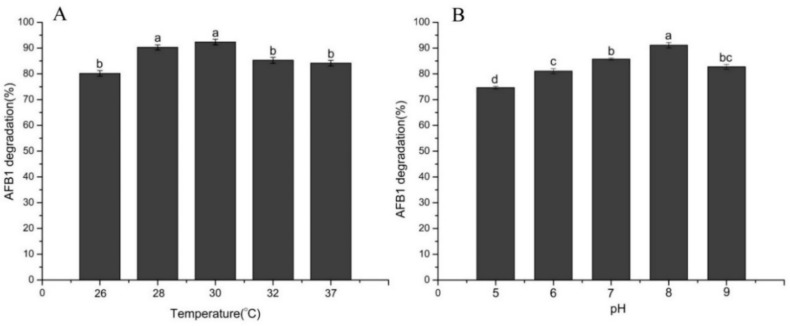
Effect of the incubation temperature (**A**) and initial pH of the medium (**B**) on AFB_1_ degradation by *B. velezensis* DY3108 after 96 h incubation. Means with different letters are significantly different according to Tukey’s test (*p* < 0.05).

The effects of temperature and culture medium pH on the degradation of AFB_1_ are shown in Figure 4. In this study, AFB_1_ was degraded by *B. velezensis* DY3108 at all incubation temperatures (Figure 4A). This may occur because strain DY3108 could produce a range of extracellular enzymes or the compatibility of the enzymes to maintain catalytic activity over a wide range of temperatures. In addition, the degradation rate reached its maximum at 30 °C when the percentage of AFB_1_ degradation was 80.19%, 90.26%, 92.36%, 85.26% and 84.13% at 26 °C, 28 °C, 30 °C, 32 °C, and 37 °C, respectively. However, the degradation rate did not show a significant difference in the range of 28–30 °C (Figure 4A). This result agrees with that of Guan et al. [24], since they point out that the degradation rates of AFB_1_ demonstrated no statistically significant difference between 20 and 30 °C when *Stenotrophomonas maltophilia* 35-3 was used to detoxify AFB_1_. Maximum degradation of AFB_1_ was also observed at 30 °C and pH 6.0 for *Rhodococcus erythropolis* ATCC 4277 to degrade, while for *Streptomyces* strains, the optimal degradation conditions were 30 °C and pH 5.0 [26]. 

The effect of the initial pH of the culture on AFB_1_ degradation showed that the degradation efficiency of AFB_1_ was sensitive to initial pH of the medium. The degradation capability decreased in parallel with a decrease in pH (Figure 4B). *B. velezensis* DY3108 gained the highest AFB1 removal efficiencies (91.10%) at pH 8, while removal efficiency 74.64% was achieved at pH of 5. This was similar to the findings of Kong et al. [27], whose results showed that pH had a positive linear effect on the degradation of AFB_1_ by *Rhodococcus erythropolis* 4.1491, and increasing the pH properly is conducive to increasing degradation. Guan et al. [28] studied the initial pH of the medium on AFB_1_ biotransformation by *Myxococcus fulvus* ANSM068 and found that a pH value between 6.5 and 7.5 favored the reaction and that the optimal pH value was 7.5.

### 2.3. AFB_1_ Degradation by Culture Supernatant of B. Velezensis DY3108

**Figure 5:**
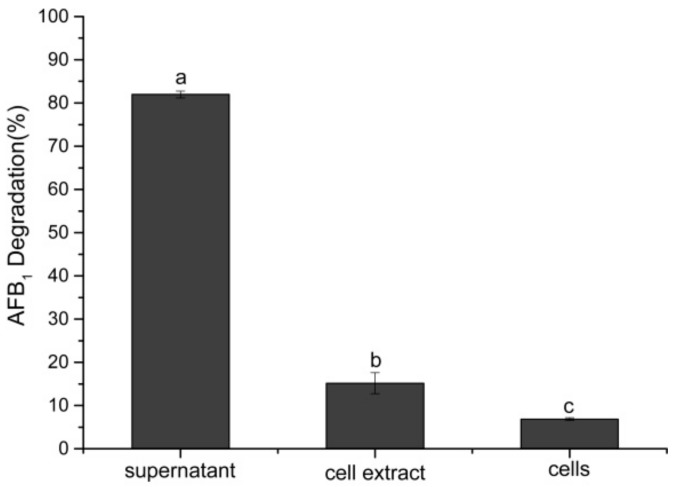
Biological degradation of AFB_1_ by the cell-free supernatant, cells, and cell extract of *B. velezensis* DY3108 at 30 °C after 72 h incubation. Values represent the mean ± SE (*n* = 3) and different letters indicate significant differences among them (*p* < 0.05).

The culture supernatant of *B. velezensis* DY3108 appeared to be more effective in the degradation of AFB_1_ than viable cells and cell lysate (*p* < 0.05). After 72 h incubation, the culture supernatant could degrade 81.97% AFB_1_ compared to 6.87% and 15.17% by viable cells and cell lysate, respectively, indicating that *B. velezensis* DY3108 degrades AFB_1_ rather than absorbing it to the cell wall (Figure 5). Rao et al. [22] previously reported that approximately 94.73% of AFB_1_ was degraded when treated with the culture supernatant of *B. licheniformis* CFR1 for 72 h. Similarly, it was also found that the cell-free supernatant of *Flavobacterium aurantiacum* can degrade 74.5% of AFB_1_ after 24 h incubation [29], while AFB_1_ degradation primarily took place in the cell-free extracts of *S. maltophilia* 35-3 with 78.7% AFB_1_ degradation after 72 h incubation with culture supernatant [24]. According to Adebo et al. [5], the degradation of AFB_1_ by extracellular elements overcomes the disadvantages of applying a whole microorganism, which may impair the organoleptic and nutritional properties of the product.

### 2.4. Effect of Temperature, pH, Time, and Metal Ions on AFB_1_ Degradation

**Figure 6:**
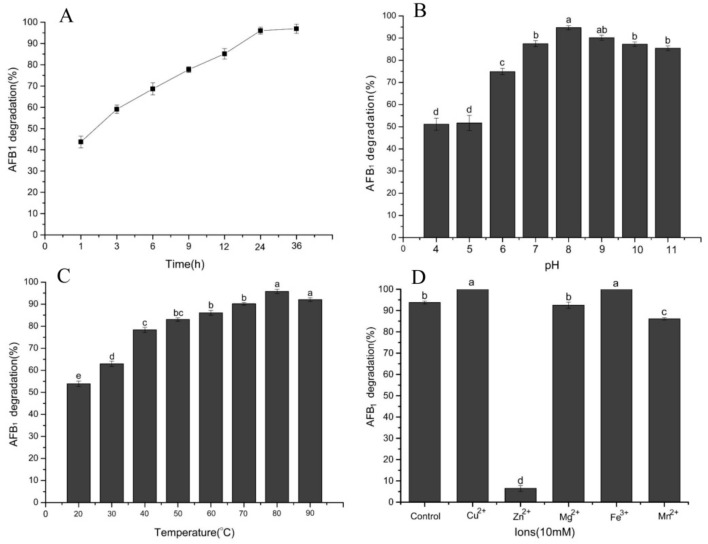
The influence of incubation conditions on the biodegradation of AFB_1_ after 24 h incubation with the cell-free supernatant of *B. velezensis* DY3108. (**A**) Influence of incubation time. (**B**) Influence of pH. (**C**) Influence of temperature. (**D**) Influence of metal ions. All of the degradation experiments were conducted at 80 °C except the temperature experiment. Values represent the mean ± SE (*n* = 3) and different letters indicate significant differences among them according to Tukey’s LSD test (*p* < 0.05).

The influence of temperature, pH, time, and metal ions on the aflatoxin B_1_-degradation activity of cell-free extracts are shown in Figure 6. As shown in Figure 6A, it was a relatively rapid and sustained degradation process for the AFB_1_, since 68.66% of AFB_1_ was removed in the initial 6 h and 96.93% was removed after 48 h incubation. Rao et al. [22] indicated that the cell-free supernatant of *B. licheniformis* CFR1 could degrade 54% of AFB_1_ in the initial 12 h and remove 93.57% after incubation for 72 h. El-Deeb et al. [30] also indicated that the degradation of AFB_1_ by *Bacillus* sp. TUBF1 was primarily in the culture supernatant, and approximately 90% of AFB_1_ was removed within the first 12 h while it was reduced to undetectable levels after 24 h. The constant increase in degradation with time indicates that the removal of AFB_1_ by the cell-free supernatant of *B. velezensis* DY3108 is an enzymatically catalyzed reaction, and cell binding plays an insignificant role in AFB_1_ reduction.

The DY3108 culture supernatant worked well over a broad pH between 4.0 and 11.0 (Figure 6B). The amount of degradation increased with increasing of the pH. The highest degradation rate (94.70%) was obtained at pH 8.0, while the lowest removal efficiency was found at pH 4 (51.16%). The effect of pH on the degradation of AFB_1_ by the culture supernatant of *S. maltophilia* demonstrated a similar trend [24], since approximately 14% of AFB_1_ was degraded at pH 4, increased to 85% at pH 8 and decreased to 70% at pH 9. In addition, it is common knowledge that enzyme had maximal activity at their optimal pH, and the value beyond the optimal range may impair their activity. However, the DY3108 culture supernatant works well over a broad range of pH (pH 6–11, >70% AFB_1_ degradation), indicating that the enzyme involved in the degradation of AFB_1_ by the DY3108 is pH stable, which is advantageous for industrial applications in the area of food safety and quality.

Most of the strains that have been reported displayed AFB_1_ degradative ability in a narrow temperature range. Sangare et al. [12] found that *Pseudomonas aeruginosa* reduce >40% of AFB_1_ between 20–65 °C, while Zhang et al. [31] indicated that *A. niger* possessed approximately 25–45% degradation ratio at 20–50 °C. It was suggested that the cell free supernatant fluid of *B. subtilis* UTBSP1 could remove 60–80% AFB_1_ at 20–40 °C [32]. Interestingly, AFB_1_ degradation studies with different incubation temperatures showed that the DY3108 culture supernatant could significantly reduce AFB_1_ content over a broad temperature range between 20–90 °C and reached the maximal level of degradation at 80 °C (Figure 6C). Guan et al. [24] reported the maximum removal efficiency of AFB_1_ by *S. maltophilia* was at 37 °C, since the *S. maltophilia* strain was isolated from the feces of the warm-blooded organism *Tapirus terrestris*. Tan et al. [33] also reported that the optimal degradation of zearalenone by *Pseudomonas otitidis* TH-N1 occurred at 37 °C. However, *Fusarium* sp. WCQ3361 metabolites displayed excellent thermostability, since temperature change (0–90 °C) did not significantly affect the AFB_1_ degradation activity [23]. Similarly, the culture supernatant of DY3108 showed excellent thermostability, since the degradation rate increased with increasing temperature up to 80 °C. The reason may be that the active constituent of the culture supernatant could work well within a wide working temperature span or several active components which are presented in supernatant and could work under different temperatures. However, more importantly, the excellent thermostability indicates that DY3108 may be an excellent potential application for the degradation of AFB_1_.

Potential effects of adding metal ions to the cell-free extracts of DY3108 on the AFB_1_ degradative ability were studied (Figure 6D). It showed that Cu^2+^ and Fe^3+^ enhanced removal efficiency, and Mg^2+^ displayed no significant effect, while Zn^2+^ strongly inhibited the degradative ability. These results suggest that Cu^2+^ and Fe^3+^ may play a role as activators or membrane stabilizers of enzyme to maintain the structural integrity and function of these proteins. The activation effect attained with Cu^2+^ is agreed with the study agrees with the results of Sangare et al. [12] who found that 10 mM of Cu^2+^ increased AFB_1_ degradation level by 29.6% using *Pseudomonas aeruginosa* N17-1, while Rao et al. [22] also indicated that Cu^2+^ and Mg^2+^ stimulated the degradation of AFB_1_ by cell-free extracts of *B. licheniformis* CFR1. However, it has been reported in *Flavobacterium aurantiacum* NRRL B-184 [29] that Mn^2+^ and Zn^2+^ can significantly decrease aflatoxin B_1_ degradation because Zn^2+^ may cause conformational changes in enzyme active sites, which leads to decreases in the affinity of AFB_1_. In addition, the effect of various metal ions on the AFB1 degradation activity of DY3108 further confirmed the enzyme’s involvement in AFB_1_ degradation.

### 2.5. Heat Treatment, SDS, and Proteinase K on AFB_1_ Degradation

**Figure 7:**
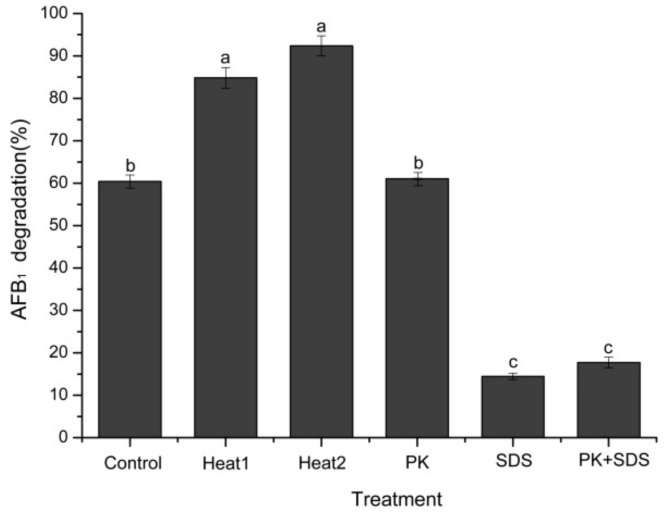
Degradation of AFB_1_ by untreated, heat-treated, proteinase K-treated, SDS-treated, SDS plus proteinase K-treated cell-free supernatant of *B. velezensis* DY3108. heat1: boiled for 30 min; heat2: autoclaved for 30 min. Different letters indicate significant differences among them according to Tukey’s LSD test (*p* < 0.05).

The AFB_1_ degradation ability of the culture supernatant significantly decreased (*p* < 0.05) after SDS and proteinase K plus SDS treatments, while the proteinase K treatment alone slightly affected the degradation activity (*p* > 0.05) (Figure 7). Similar results were also found in other aflatoxin B1 degrading bacteria, such as *Mycobacterium fluoranthenivorans*, *P. aeruginosa*, and *R. erythropolis* [11,13,24]. It showed that proteinase K-treated culture supernatant displayed low levels of AFB_1_ degradation activity, but when subjected to proteinase K plus SDS treatment, the AFB_1_ degradation activity was completely destroyed [18]. More importantly, when the supernatant was treated with heat (boiled or autoclaved for 30 min), the complete loss of AFB_1_ degradative activity was not detected, but a stimulated degradative ability was observed, which further proved that the enzymes involved in AFB_1_ degradation possessed excellent thermostability and high activity. The observed thermal activation of the enzymes was also found in other microorganisms. Sangare et al. [12] reported that the AFB_1_ degradation rate decreased significantly when the cell-free supernatant of *P. aeruginosa* N17-1 was treated with proteinase K plus SDS. However, when supernatant was subjected to heat treatment (boiling water bath for 10 min), AFB_1_ degradation activity was stimulated by thermal activation (increased by 3.49%). Kavitha et al. [34] isolated an antifungal protein with robust thermal stability from *Bacillus polymyxa* strain VLB16, which still maintained activity after sterilization (15 min at 121 °C). In addition, Wang et al. [23] found that the metabolites of *Fusarium* sp. WCQ3361 exhibited high thermal stability and 99.40% residual activity to degrade AFB_1_ was retained even after boiling for 10 min. Since mesophilic enzymes are often failed to endure the harsh reaction conditions required in industrial processes, it is extremely beneficial that thermostable enzymes may provide robust and efficient catalyst substitutes that can withstand the harsh reaction conditions required in industrial processes [35]. Therefore, the excellent thermostability of the enzyme’s involvement in AFB_1_ degradation by DY3108 offers a potentially valuable solution to remove aflatoxin from human diet and animal feed.

### 2.6. Cytotoxicity Study

**Figure 8:**
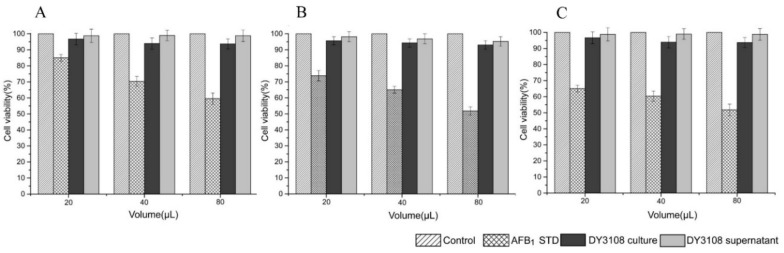
Cell viability (percent of viable cells) of the DY3108 culture and the culture supernatant bio-transformed products (DY3108 supernatant) with different incubation times: (**A**) 24 h, (**B**) 48 h, and (**C**) 72 h.

The cytotoxicity and mutagenicity of the degradation products of mycotoxins should not be neglected since certain degradable products may be toxic like their parent compounds [1]. Therefore, it is indispensable to clarify the toxicity of the AF biodegradation products by DY 3108 in comparison to the parent compound AFB_1_. In the present study, DMSO exposure alone at the test concentration did not significantly affect cell viability and the cytotoxicity of the biodegradation extract against human lymphocytes was evaluated via the MTT assay for 24, 48 and 72 h (Figure 8A–C). It is generally observed that the percentage of cell viability was related inversely to the AFB_1_ concentration. In addition, all of the bio-transformed extracts (culture and culture supernatant treatment) had more than 90% cell viability, comparable with those of the control sample. The corresponding diminution in the cytotoxic effect of AFB_1_ biodegradation extracts suggested that the AFB_1_-lactone ring had been cleaved and modified [36]. Similarly, high levels of cell viability of human lymphocytes were also reported by Adebo et al. [5] who found that human lymphocytes retained >90% viability when cells were exposed to AFB_1_ biodegradation extracts of *Pontibacter* sp. VGF1. Since the degradable or transformed products produced by DY3108 were less toxic than the parent compound AFB_1_, *B. velezensis* DY3108 has potential for use as a bio-detoxification agent for AFB_1_ in food commodities.

### 2.7. Analysis of AFB_1_ Degradation Products Using LC-MS

**Figure 9:**
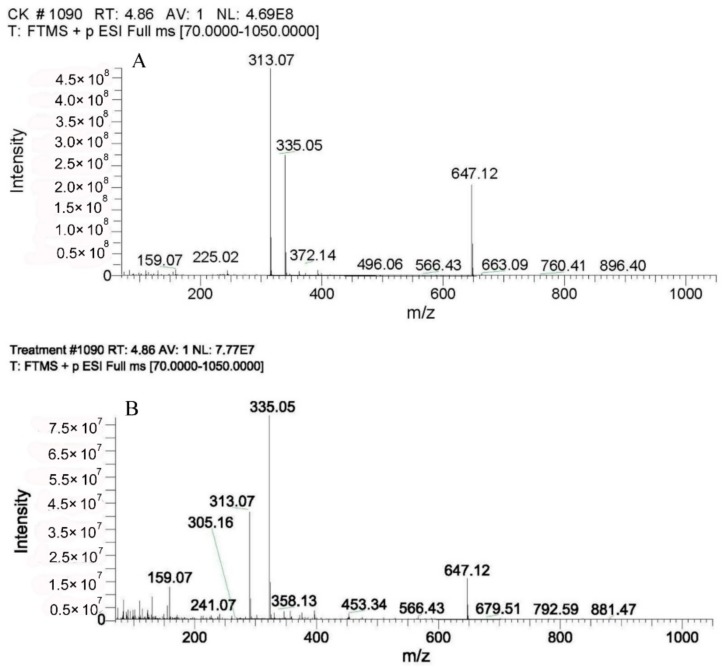
Full mass spectra of AFB_1_: (**A**) AFB_1_ in nutrient broth (NB) culture medium (CK) (**B**) AFB_1_ in NB culture medium treated with *B. velezensis* DY3108.

As shown in Figure 9A, molecular ions with *m*/*z* = 313, 335, and 647, which were specific for AFB_1_, presented in the full mass spectrum of AFB_1_ in nutrient broth (NB) medium (CK). From the MS analysis of the DY3108-treated samples it became evident that AFB_1_ was still present in the biodegradation extracts, but the incidence of its molecular peak declined sharply compared with that in the non-inoculated control (Figure 9B), which further affirmed the degradation of AFB_1_ by DY3108. However, in this study, it failed to identify any breakdown product of AFB_1_ by DY3108. Similar results have been reported by Farzaneh et al. [32], Rao et al. [22] and Sangare et al. [12]. It showed that *Bacillus subtilis* JSW-1 can degrade AFB_1_ effectively. However, no biodegradation products can be clearly identified by the liquid chromatography-mass spectrometry (LC-MS) analysis [14]. One reason for this is that AFB_1_ was most probably decomposed to certain components whose chemical characteristics were different from that of parent AFB_1_. 

## 3. Conclusions

In summary, a *Bacillus* strain that can degrade more than 90% AFB_1_ was isolated and identified as *Bacillus velezensis* DY3108. More importantly, to the best of our knowledge, this is the first study to demonstrate more than 90% degradation of AFB_1_ by a *B. velezensis* strain. It showed that the degradation capability was attributed to extracellular proteins or enzymes present in the culture supernatant. The degraded products differed chemically from AFB_1_ and showed significantly lowered cytotoxicity. In addition, these extracellular proteins or enzymes possess a broad reaction temperature range and pH tolerance, as well as excellent thermostability which will help them withstand the harsh conditions in food processing. Therefore, the enzymes or proteins in the supernatant are promising new agents for AF biodegradation from the human diet and animal feed. However, further research such as adequate purification and characterization of the enzymes or proteins in the supernatant are needed to exploit the probable use of the *B. velezensis* DY3108 in food and feed. Furthermore, it should be noted that the bacterial strains isolated from coumarin medium plates might have taken up coumarin from the original screen and that coumarin may have certain influence on aflatoxin degradation. Therefore, further research could focus on determination of coumarin content in bacterial strains (using HPLC, MS, etc.) to determine whether coumarin was taken up by bacterial strains and thus participated in degradation of aflatoxin. Meanwhile, more research is needed to identify coumarin-isolates interactions, and elucidate the mechanisms of coumarin uptake or degradation, to provide new insights into the aflatoxins bioremediation.

## 4. Materials and Methods

### 4.1. Chemicals and Medium

AFB_1_ standard reagent was purchased from Sangon Biotech Co., Ltd. (Shanghai, China) and diluted into methanol (HPLC grade) to prepare AFB_1_ stock solution (at 10 ppm). All other analytical grade reagents and HPLC grade solvents were purchased from Sinopharm Chemical Reagent Co., Ltd. (Shanghai, China). Wild-type *A. flavus* strain 3.6305 and *A. parasiticus* strain 3.6155 were purchased from the China General Microbiological Culture Collection Center (CGMCC), and cultured on potato dextrose agar (PDA) medium at 28 °C. 

### 4.2. Isolation of AFB_1_ Degrading Bacteria

#### 4.2.1. Isolation of Microorganisms

Six soil samples were sampled from cropland of NongKang state farm, Bengbu, Anhui Province, China. The primary screening for microorganisms that able to remove or degrade AFB_1_ was carried out as described by Guan et al. [24] with small modifications. 

Briefly, one gram of the soil sample was diluted in 9 mL sterile water and kept at room temperature under continuous shaking (200 rpm) for 6 h. And then, the samples were serially diluted (10^1^–10^9^) in sterilized distilled water. Aliquots (100 μL) of each dilution was spread on coumarin medium plates [24] and incubated at 30 °C for 4–5 days until visible colonies appeared on plates. The single colonies were picked from the plates and then streak on fresh plates. Repeat the above process 4–5 times and the pure isolates were finally preserved on LB agar and tested for AFB_1_-degradation activity.

#### 4.2.2. Secondary Screening

AFB_1_-degradation activities of the chosen isolates were performed as follows: Briefly, each isolate was grown in nutrient broth (NB) (Oxoid Ltd., Hants, UK) at 30 °C for 20 h in a rotary shaker incubator (180 rpm). One hundred microliters AFB_1_ stock solution (10 ppm) was mixed with 1.9 mL culture broth to acquire the desired concentration (500 ppb), and then cultured in a rotary shaker incubator (180 rpm) at 30 °C for 4 days. AFB_1_ standard with sterile NB was used as—control. When the reaction is completed, the suspension was centrifuged at 8000× *g* for 20 min to recover the cell-free supernatant. For quantitation of residual AFB_1_, samples were extracted four times with chloroform, evaporated, and dissolved in 1 mL-methanol (HPLC grade), followed by filtering through 0.22 µm Nylon filter (Sangon Biotech Co., Ltd., Shanghai, China) and preserved at 4 °C until analysis.

### 4.3. Analysis of Residual AFB_1_

The determination of AFB_1_ in samples was conducted by HPLC using LC-8A (Shimadzu, Kyoto, Japan) with a Varian C18 column (5 μm, 250 mm × 4.6 mm) and AOAC method as previously described by Xia et al. [14] with slight modifications. A water/acetonitrile/ methanol mix (1.0:1.5:1.5, *v*/*v*/*v*) was used as mobile phase, and the flow rate was 1 mL/min. AFB_1_ concentration was quantitatively determined using a fluorescence detector, and the detection wavelengths for excitation and emission were 365 nm and 418 nm, respectively. The limit of detection (LOD) was 5 ng/mL AFB_1_, and the limit of quantitation (LOQ) was 16 ng/mL AFB_1_ under the experimental conditions used.

In both cases, the AFB_1_ removal efficiency was calculated by following formula:

(1 − AFB_1_ peak area in treatment/AFB_1_ peak area in control) ×100 (1)

### 4.4. Identification of the Isolate “DY3108”

Standard physical and biochemical tests of the DY3108 isolate were conducted using standard methods [37]. For the 16S rRNA gene sequence analysis, genomic DNA of strain DY3108 was isolated using an EasyPure Bacteria Genomic DNA Kit (TransGen Biotech Co., Ltd., Beijing, China). A 1471-bp 16S rRNA gene fragment was subsequently amplified with the 27F and 1492R primers [38] and sequenced as described by Huang et al. [39]. The sequence was then aligned against those found in the NCBI database, and on the EzBioCloud server [40] using the Basic Local Alignment and Search Tool (BLAST) algorithm. These sequences were then utilized to construct a phylogenetic tree using MEGA 7 with the maximum likelihood (ML) method [41]. The sequence has been deposited to the GenBank database under the accession number MG839279. 

### 4.5. In Vitro Anti-Aflatoxigenic Effect

Biocontrol activity assays of DY3108 against AFB_1_ produced by *A. flavus* were conducted in 250 mL flasks as described by Gong et al. [42]. Briefly, 20 g of maize grains was added into 250 mL flasks and autoclaved at 121 °C for 30 min. After cooling to room temperature, maize grains in each flask were inoculated with 1 mL of 5 × 10^5^ CFU/mL suspension AF conidia. The samples were divided equally into two parts: half was challenged with 10 mL DY3108 culture (grown on NB medium with 10^8^ CFU/mL), and the other half was treated with 10 mL NB medium as a control. After 7 days incubation at 28 °C, samples in each treatment were collected and dried at 90 °C for 5 days. Then the dried samples were ground to a fine powder and subjected to aflatoxin analyses.

### 4.6. Effects of Culture Conditions of AFB_1_-Degrading Bacteria on Biodegradation

The effects of different culture conditions on AFB_1_-degradation activity of the DY3108 were conducted as follows: incubation temperature (26, 28, 30, 32, and 37 °C), culture medium pH (5, 6, 7, 8, and 9), and incubation time (6, 12, 24, 48, 72, and 96 h). The AFB_1_ concentration in the culture medium was 500 ppb. The reactions were manipulated in a rotary shaker incubator (180 rpm), and residual AFB_1_ was detected using HPLC as described previously.

### 4.7. AFB_1_ Degradation by Cells, Culture Supernatant, and Cell Lysate

The biodegradation of AFB_1_ by cells, cell-free supernatant, and cell lysate were conducted as described by Rao et al. [22] with slight modifications. Briefly, isolate DY3108 was grown in NB medium for 20 h at 30 °C. The cells were then harvested by centrifugation for 20 min at 8000× *g*. The supernatant was immediately filtered through 0.22 μm filter for further experiments, while the pellet was washed three times with sterile MilliQ water and dissolved in Phosphate buffer (0.1 M, pH 7.0). Then, the cell suspension was divided into two fractions: in one the cells were suspended in Phosphate buffer without any treatment (namely whole bacterial cell), and in the other, the cells were disintegrated using an ultrasonicator (Ningbo Scientz Biotechnlogy Co., Ltd., Ningbo, China), and centrifuged at 10,000× *g* for 40 min at 4 °C. After centrifugation, the obtained supernatant was filtered through 0.22 μm filter (namely cell lysate) and used in subsequent AFB_1_ degradation experiments. For AFB_1_ degradation, AFB_1_ (final concentration 500 ppb) were treated with the culture supernatant, whole bacterial cell, and cell lysate respectively, and then incubated at 30 °C for 72 h. Non-incubated cultures (NB + PBS) with AFB_1_ was used as control, and conducted as described previously.

### 4.8. Optimization of Conditions for Maximum Degradation of AFB_1_

The culture supernatant of strain DY3-108 was prepared as described previously. AFB_1_ (500 ppb) was incubated with the supernatant at 1, 3, 6, 9, 12, 24, and 36 h, and the analysis was carried out. AFB_1_ treated with uninoculated NB medium served as the control. In order to investigate the influence of temperature on the degradative efficiency, the supernatants combined with AFB_1_ were incubated at 20, 30, 37, 50, 60, 70, 80, and 90 °C for 24 h. The pH influence on degradation was conducted by adjusting the culture supernatant to pH 4–5 with citrate buffer, pH 6–8 with phosphate buffer, pH 9 with sodium carbonate buffer, and pH 10–11 with sodium carbonate/sodium bicarbonate buffer. Blank of uninoculated NB medium with a corresponding pH used as the control. The effect of various metal ions on the degradation was obtained by adding 10 mM of Mg^2+^ (MgCl_2_), Cu^2+^ (CuSO_4_), Mn^2+^ (MnCl_2_), Zn^2+^ (ZnSO_4_), or Fe^3+^ (FeCl_3_) to the supernatant and incubated with AFB_1_, while in the control the supernatant was replaced by NB. All of the AFB_1_ degradation experiments were conducted at 80 °C except for the temperature experiment.

### 4.9. Heat, SDS, and Proteinase K Treatment on AFB_1_ Degradation

In order to study the influence of heat treatment on degradation efficiency, the supernatant fractions were boiled for 30 min or placed in an autoclave for 30 min. Meanwhile, the effect of proteinase K and sodium dodecyl sulfate (SDS) treatments on the degradation ability was investigated by exposing them to 1 mg/mL proteinase K and 1% SDS, respectively, at 30 °C in the dark for 24 h as described by [21]. Blank of uninoculated NB medium and untreated supernatant culture were used as control. The extraction and the analysis of residual AFB_1_ were conducted as described previously.

### 4.10. Cytotoxicity Studies

Lymphocyte cells were purchased from Shanghai JRDUN Biotechnology Co., Ltd. (Shanghai, China) and cultured according to the method reported [5]. After the completion of culture, final cell concentrations were determined, and cells showing 100% vitality were used in subsequent trials.

The MTT assay was performed as described by Samuel et al. [2] and Adebo et al. [13] with minor modifications. Briefly, for preparing the MTT (3-(4,5-dimethylthiazol-2-yl)-2,5-diphenyltetrazolium bromide) assay, cell suspension (180 μL) which was stimulated with 10 mg/mL phytohemagglutinin-p (PHA-p) was pipetted into 96-well plate. Then the cells were respectively dealt with different volumes (20, 40 and 80 μL) of AFB_1_ standard solution (2.5 ppm, redissolved in DMSO with culture media), extracts of AFB_1_ degradation products (previously evaporated to dryness and redissolved in DMSO with culture media) as well as the DY3108 culture. Exactly 160 μL of culture media was then added to each well and cultured at 37 °C in a 5% CO_2_ humidified incubator for 24, 48 and 72 h respectively. After incubation, 30 μL of MTT solution (5 mg/mL in 0.14 M PBS) was added into each well and continued to be cultured for 3 h under the same conditions. Cells were exposed to the corresponding concentration of DMSO alone to assess the cytotoxic effects of DMSO on cell viability. Untreated cells were used as a negative control.

Then, 50 μL of dimethylsulfoxide (DMSO) was pipetted into each well and an additional 2 h incubation was performed. After incubation, the absorbance was determined using a microplate reader that was set at a wavelength of 560 nm. Percentage of viable cell can be expressed as (AS/AC)/100, where AS and AC are the absorbance of the treated and untreated (control) samples.

### 4.11. Analysis of AFB1 Degradation Products by LC-MS

LC-MS/MS method was applied to analyze the AFB_1_ degradation products by using Thermo LXQ liquid chromatography coupled with ion trap mass spectrometry (Thermo Scientific, Waltham, MA, USA). Liquid chromatography was achieved using a Shim-pack VPODS C18 column (100 × 2.1 mm). The mobile phase used in this study comprised of 0.1% formic acid water and 0.1% formic acid acetonitrile, and the flow rate was 1 mL/min. MS was performed as follows for the positive ion mode: m/z range, 50–800; ion spray voltage 4.5 kV, sheath gas flow rate 36 L/min, auxiliary gas flow rate 10 L/min, and capillary temperature 300 °C. 

### 4.12. Statistical Analysis

All analyses were conducted in triplicate, and the values represent the average of the measurements conducted from three independent assays and are expressed as the mean ± standard error of the mean (SEM). The data were further analyzed using an ANOVA at a 95% confidence level following Tukey’s test (SPSS 18.0, IBM, Somers, NY, USA).

## Figures and Tables

**Table 1:** Aflatoxin B_1_ inhibition in maize containing co-cultures of DY3108 and *Aspergillus flavus.*

	Conc. of AFB_1_ (μg/g)	Aflatoxin B_1_ Inhibition
Maize grains + NB	ND ^1^	/
Maize grains + DY108	ND ^1^	/
Maize grains + *A.flavus*	4.23 ± 0.23 a	/
Maize grains + *A.flavus* + DY108	1.42 ± 0.21 b	66.43%

^1^ ND: not detected.

## Supplementary Materials

The following are available online at http://www.mdpi.com/2072-6651/10/8/330/s1, Table S1: Physiological and biochemical characteristic of the bacterial isolate DY3108.
