# Supplementary material for: Biological Degradation of Aflatoxin B1 by Cell-Free Extracts of Bacillus velezensis DY3108 with Broad PH Stability and Excellent Thermostability
Source: Toxins (Basel). 2018 Aug 14;10(8):330. doi: 10.3390/toxins10080330 (PMC6116002; doi:10.3390/toxins10080330)
Supplement: Supplementary file 1 [file toxins-10-00330-s001.pdf]

Supplementary Materials: Biological Degradation of Aflatoxin B_1 by Cell-Free Extracts of *Bacillus velezensis* DY3108 with Broad PH Stability and Excellent Thermostability

Xian Shu, Yuting Wang, Qing Zhou, Minghao Li, Hao Hu, Yuhan Ma, Xue Chen, Jun Ni, Weiwei Zhao, Shengwei Huang and Lifang Wu

Table S1. Physiological and biochemical characteristic of the bacterial isolate DY3108.

Characteristic	Result	Characteristic	Result
Gram-staining	+	Assimilation of	
Pigmentation<br>Oxidase	Creamy white	Glycogen	+
Motility	+	Lactose	+
Oxidase	+	Melibiose	-
Catalase	+	D-fructose	+
V-P test	+	D-Raffinose	+
Indole test	-	D-arabitol	+
Nitrate reduction	+	Mannitol	+
Urease	-	inositol	+
Hydrolysis of		D-xylose	+
casein	+	β-methyl-xyloside	+
Gelatin	+	Growth in 5% NaCl	+
Growth at 5°C	-	Growth in 10% NaCl	+
Growth at 42°C	+		
